# CRISPR/Cas9‐mediated deletion of *Fam83h* induces defective tooth mineralization and hair development in rabbits

**DOI:** 10.1111/jcmm.17597

**Published:** 2022-10-27

**Authors:** Yuxin Zhang, Jie Yang, Haobin Yao, Zhongtian Zhang, Yuning Song

**Affiliations:** ^1^ Key Laboratory of Zoonosis Research Ministry of Education College of Veterinary Medicine Jilin University Changchun China

**Keywords:** abnormal mineralization, amelogenesis imperfecta, CRISPR/Cas9 system, hair defects, rabbits

## Abstract

Family with sequence similarity 83 members H (*Fam83h*) is essential for dental enamel formation. *Fam83h* mutations cause human amelogenesis imperfecta (AI), an inherited disorder characterized by severe hardness defects in dental enamel. Nevertheless, previous studies showed no enamel defects in *Fam83h*‐knockout/lacZ‐knockin mice. In this study, a large deletion of the *Fam83h* gene (900 bp) was generated via a dual sgRNA‐directed CRISPR/Cas9 system in rabbits. Abnormal tooth mineralization and loose dentine were found in homozygous *Fam83h* knockout (*Fam83h* ^−/−^) rabbits compared with WT rabbits. In addition, reduced hair follicle counts in dorsal skin, hair cycling dysfunction and hair shaft differentiation deficiency were observed in *Fam83h* ^−/−^ rabbits. Moreover, X‐rays and staining of bone sections showed abnormal bending of the ulna and radius and an ulnar articular surface with insufficient trabecular bone in *Fam83h* ^−/−^ rabbits. Taken together, these data are the first report of defective hair cycling, hair shaft differentiation and abnormal bending of the ulna and radius in *Fam83h* ^−/−^ rabbits. This novel *Fam83h* ^−/−^ rabbit model may facilitate understanding the function of *Fam83h* and the pathogenic mechanism of the *Fam83h* mutation.

## 1 INTRODUCTION

*Fam83h* (family with sequence similarity 83, member H; OMIM *611927) is a protein‐coding gene that plays an essential role in the structural organization and calcification of developing enamel,^1^ and the regulation of the keratin cytoskeleton by recruiting Casein kinase 1 to keratin filaments.^2^, ^3^, ^4^ Previous studies showed that the nonsense, missense or deletion mutations of the fifth exon of the *Fam83h* gene will result in the production of an abnormal protein that induces pathological changes.^2^, ^5^ Moreover, the eight members of the FAM83 family of proteins are conserved in vertebrates but are poorly characterized. They share a conserved N‐terminal DUF1669 (domain of unknown function 1669) domain of ~300 amino acids, but each member has a unique C terminus of variable length.^3^ At present, it has been reported that *Fam83h* affects keratin cytoskeleton and desmosome formation by recruiting casein kinase I (CK‐1) to keratin, since its N‐terminus and C‐terminus can bind CK‐1 and keratin, respectively. Hence, the combination of CK‐1 and keratin has an effect on desmosome formation,^6^ and it may have vital effects on the homeostasis of epidermal development and the regulation of the hair cycle and hair shaft development.^6^

A genome‐wide search identified *Fam83h* as one of the major genes with a role in causing amelogenesis imperfect (AI).^2^ AI is a hereditary dental condition showing enamel abnormalities in both primary and permanent teeth. AI exhibits a prevalence as high as one in 700 in some populations.^7^ Prior studies found that AI comprises a group of inheritable congenital genetic disorders characterized by dental enamel malformation, which can be categorized into three main types: hypoplastic, hypocalcifed and hypomatured.^8^ Although the mutation of *Fam83h* contributes to AI in clinical, its actual functions, pathogenesis and association with enamel defects are still unknown.

It is reported that most of AI patient exhibited yellow‐brown teeth, localized enamel defects and sustained defective mineralization.^9^ But, *Fam83h*‐knockout/lacZ‐knockin mice^2^ failed to recapitulate the phenotype of human enamel defects; there is no significantly difference of the thickness, density, hardness, morphology and prism patterns between *Fam83h* mutated and WT mice.^2^ Thus, *Fam83h* has debatable effects on the development of teeth and the formation of enamel in mice model. Therefore, to further investigate the function of *Fam83h* and precisely recapitulate human AI, the new animal models are needed in the further study.

Of note, rabbits are considered as a better animal models than mice in recapitulating some human diseases because rabbits have greater similarity in terms of physiology, anatomy and genetics to humans than mice.^10^ Here, we generated *Fam83h* knockout (KO) rabbits using the CRISPR/Cas9 system, the abnormal dental mineralization, hair cycling disorder and hair shaft differentiation deficiency were determined in those *Fam83h* ^−/−^ rabbits. In conclusion, this is the first report of defects in hair cycling and hair shaft differentiation in the dorsal skin of *Fam83h* ^−/−^ rabbits. Thus, the present study provides further insight into the relationship between *Fam83h* mutations and AI and also a novel animal model for delayed hair cycling and defective hair shaft differentiation for the pre‐clinical study in the future.

## 2 MATERIALS AND METHODS

### 2.1 Animals and ethics statement

New Zealand rabbits used in this study were obtained from the Laboratory Animal Centre of Jilin University (Jilin, China). All animal studies were conducted according to experimental practices and standards approved by the Animal Welfare and Research Ethics Committee at Jilin University (Jilin, China).

### 2.2 Single guide RNA design and vector construction

The 3xFLAG‐NLS‐SpCas9‐NLS vector (ID 48137; Add gene) was linearized with NotI, and it was in vitro transcribed using the mMessage mMachine SP6 kit (ThermoFisher Scientific) and the RNeasy Mini kit (Qiagen) according to the manufacturer's instructions. The design of targeted single‐guide RNAs (sgRNAs) has been described previously.^11^ Complementary oligo sgRNAs were cloned into a Puc57‐T7‐sgRNA cloning vector (Addgene ID 51306) using BbsI restriction sites. Plasmids were extracted with the EZgene™ Plasmid Miniprep Kit (PD1211, Biomiga) for use.

The Puc57‐T7‐sgRNA vector was amplified by PCR using the T7 primers (T7‐F: 5'‐GAAATTAATACGACTCACTATA‐3′ and T7‐R:5‐AAAAAAAGCACCGACTCGGTGCCAC‐3′). Then, PCR products were in vitro transcribed using T7 RNA polymerase (Beijing T&L Biological Technology Co., Ltd) and purified using a miRNeasy Mini kit (Qiagen) according to the manufacturer's instructions.

### 2.3 Embryo microinjection and embryo transfer

The embryo microinjection and embryo transfer procedures were as previously described.^10^ In brief, female New Zealand white rabbits at 6–8 months of age were superovulated by 6 treatment with 50 IU of follicle‐stimulating hormone (FSH) at intervals of 12 h, then they were mated with male rabbits, and injected with 100 IU human chorionic gonadotropin (HCG). At 18 h post‐HCG injection, female rabbits were euthanized, and the oviducts were flushed with 5 ml Dulbecco's phosphate‐buffered saline – bovine serum albumin solution (DPBS‐BSA) to collect zygotes. Furthermore, a mixture of Cas9 mRNA (200 ng/μl) and sgRNA (50 ng/μl) was microinjected into the cytoplasm of rabbit zygotes. The injected embryos were transferred into Earle's balanced salt solution (EBSS) and placed at 38.5°C and 5% CO_2_ for short‐term culture. Finally, 30–50 injected zygotes were transferred into the oviduct of recipient rabbits.

### 2.4 Mutation detection in pups by PCR

The DNA of *Fam83h*‐KO and WT rabbits was isolated from a small piece of ear tissue using the TIANamp Genomic DNA kit (DP304‐03, Tiangen) according to the manufacturer's instructions. Mutation detection was performed by PCR using the following primers (Table S1). PCR products were gel purified and cloned into a pGM‐T vector (VT202‐01, Tiangen). At least 10 positive plasmid clones were sequenced (Sangon Biotech) and analysed using DNAman.

### 2.5 Off‐target analysis

Potential off‐target sequences were selected by using the CRISPR Design tool (http://crispr.mit.edu/). PCR products were subjected to T7 endonuclease I assay (EN303‐01, Vazyme Biotech) and Sanger sequence analysis (Sangon Biotech). The primers are listed in Table S2.

### 2.6 T7EI cleavage assay

The T7EI assay was performed as described previously.^12^ Briefly, PCR products were purified using a TianQuick Midi purification kit (DP204‐03, Tiangen), then denatured and annealed in NEBuffer 2 (NEB) using a thermocycler. Hybridized PCR products were digested with T7 endonuclease I (NEB, M0302L) for 30 min at 37°C and subjected to 2% agarose gel electrophoresis.

### 2.7 RNA extraction and qRT‐PCR

Total RNA was isolated from rabbit dorsal skin of WT and *Fam83h*‐KO rabbits using TRNzol‐A^+^ reagent (Tiangen) and then treated with DNase I (Fermentas). Primers used for qRT‐PCR are shown in Table S3. qRT‐PCR was performed using a SYBR PrimeScript RT‐PCR kit (Bioer Technology), and the 2^−ΔΔCT^ formula was used to analyse gene expression. *Gapdh* was used as a reference gene. All experiments were repeated three times for each gene. The data are expressed as the mean ± SEM.

### 2.8 Histological analysis

Haematoxylin and eosin (H&E) and Masson's trichrome staining were performed as previously described.^10^, ^13^ Briefly, the tissues were collected from *Fam83h*‐KO and WT rabbits and fixed in 4% paraformaldehyde at 4°C. Then, the tissue was dehydrated in increasing concentrations of ethanol (70% for 6 h, 80% for 1 h, 96% for 1 h and 100% for 3 h), cleared in xylene and embedded in paraffin for histological analysis under a light microscope.

For von Kossa staining, renal tissue sections were treated with 5% silver nitrate solution and exposed to incandescent light for 1 h. Then these were treated with 5% sodium thiosulfate for 1 min, and calcium salts were often stained black or brown‐black.

### 2.9 X‐ray absorptiometry

X‐ray radiography scans of the femur were taken using a YEMA Radiography System with a digital camera (Varian) attached to an X‐ray radiographer (Rotanode). Images were taken at 50 KV with 3 mAs exposure.

### 2.10 Serum biochemistry analysis

WT and *Fam83h*‐KO rabbit blood was collected at 15 months old. Serum samples were obtained by centrifugation. Serum phosphorus levels were measured using a Phosphorus Assay kit (Changchun Huili) with the peacock chlorine colorimetric method. Serum ALP levels were assayed using an ALP Assay kit (Changchun Huili) with the disodium phenyl phosphate method. Serum creatinine was measured using a Creatinine Assay kit (Changchun Huili) with the picric acid method. Serum BUN was measured using a BUN Assay kit (Changchun Huili) with the urease indoxyl method.

### 2.11 Scanning electron microscopy

Scanning electron microscopy was performed as previously described.^7^ Hair samples from the dorsal skin of *Fam83h*‐KO and WT rabbits were attached to specimen stubs using double stick conductive tabs and sputter‐coated with gold using a polaron scanning electron microscope E‐1010 (Hitachi). Samples were imaged using an S‐3400 N scanning electron microscope (Hitachi). The length of the hair was measured with a Vernier calliper.

### 2.12 Statistical analyses

Data are expressed as the mean ± SEM, with at least three individual measurements in all experiments. The data were analysed by one‐way ANOVA using GraphPad Prism 6.0 software. A probability of *p* < 0.05 was considered statistically significant.

## 3 RESULTS

### 3.1 Generation of *Fam83h*‐KO rabbits via the CRISPR/Cas9 system

**FIGURE 1:**
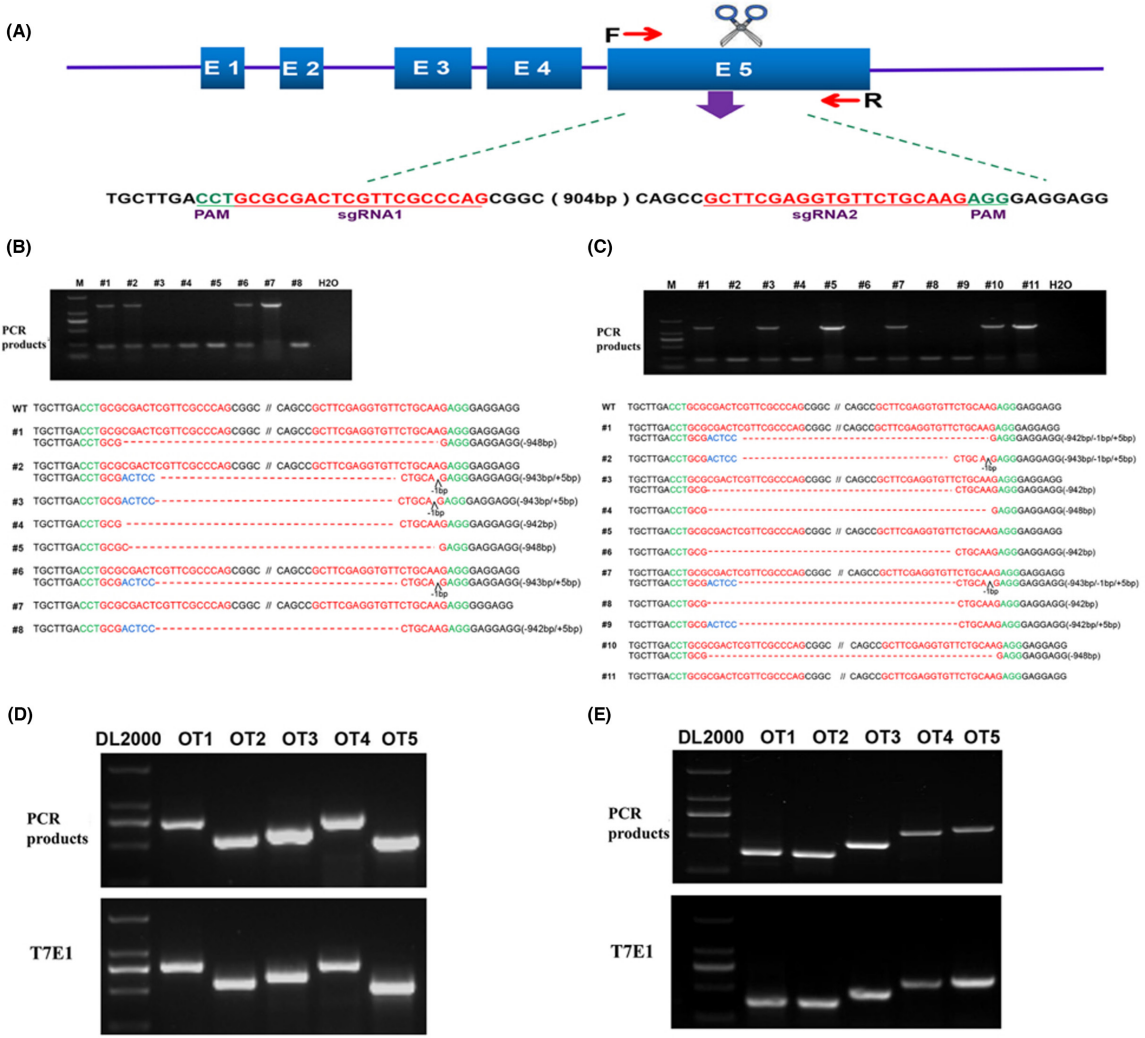
Generation of *Fam83h*‐KO rabbits by the CRISPR/Cas9 system. (A) Schematic diagram of two sgRNA target sites located in exon 5 of the rabbit *Fam83h* locus. *Fam83h* exons are indicated by blue rectangles; sgRNA target sites are highlighted in red; PAM sites are underlined and highlighted in green; (B,C) Founder (B) and F2 offspring (C) mutation detection by Sanger sequencing.; WT, wild‐type control. sgRNA target sites are highlighted in red; PAM sites are highlighted in green;insertions are shown in blue; deletions (−) are shown. (D) T7E1 cleavage analysis of seven potential off‐target sites (POTS) for sgRNA1. M, DL2000 (E). T7E1 cleavage analysis of seven potential off‐target sites (POTS) for sgRNA2, M, DL2000

To disrupt the function of the *Fam83h* gene in rabbits, a pair of sgRNAs targeting the fifth exon of rabbit *Fam83h* was designed (Figure 1A, Table S1). Mixed Cas9 mRNA and sgRNAs were co‐injected into rabbit zygotes. A total of 46 injected zygotes were transferred into the oviducts of two surrogate rabbits, and 8 live pups were obtained. As shown in Table 1 and Figure 1B, 7 out of 8 (87.5%) pups carried the *Fam83h* mutation, and 4 pups (57.14%) carried biallelic *Fam83h* mutations.

**TABLE 1:** Summary of the *Fam83h* KO rabbits generated by CRISPR/CAS9

Recipients	gRNA/Cas9 (ng/μl)	Embryos transferred	Pregnancy	Pups obtained (% transferred)	Pups with mutations (% pups)	Biallelic modified (% pups)
1	40/200	19	Yes	5 (26.3%)	4 (80%)	2 (50%)
2	40/200	27	Yes	3 (11.1%)	3 (100%)	2 (66.7%)
Total		46	100%	8 (18.7%)	7 (90%)	4 (58.35%)

To obtain a sufficient number of rabbits for detailed phenotypic characterization of the *Fam83h* mutation, F1 and F2 individuals were obtained by brother–sister breeding. The genotypes of F2 *Fam83h* KO rabbits were confirmed by PCR and Sanger sequence analysis (Figure 1C), which demonstrated that large‐fragment deletions of the *Fam83h* gene were heritable in the rabbits.

To examine the off‐target effects in *Fam83h*‐KO rabbits, the PCR products of ten potential off‐target sites were subjected to Sanger sequencing and T7E1 cleavage assays, and no off‐target mutations were detected at these sites in the *Fam83h*‐KO rabbits (Figure 1D,E).

### 3.2 Dental phenotype analysis of *Fam83h* ^−/−^ rabbits

**FIGURE 2:**
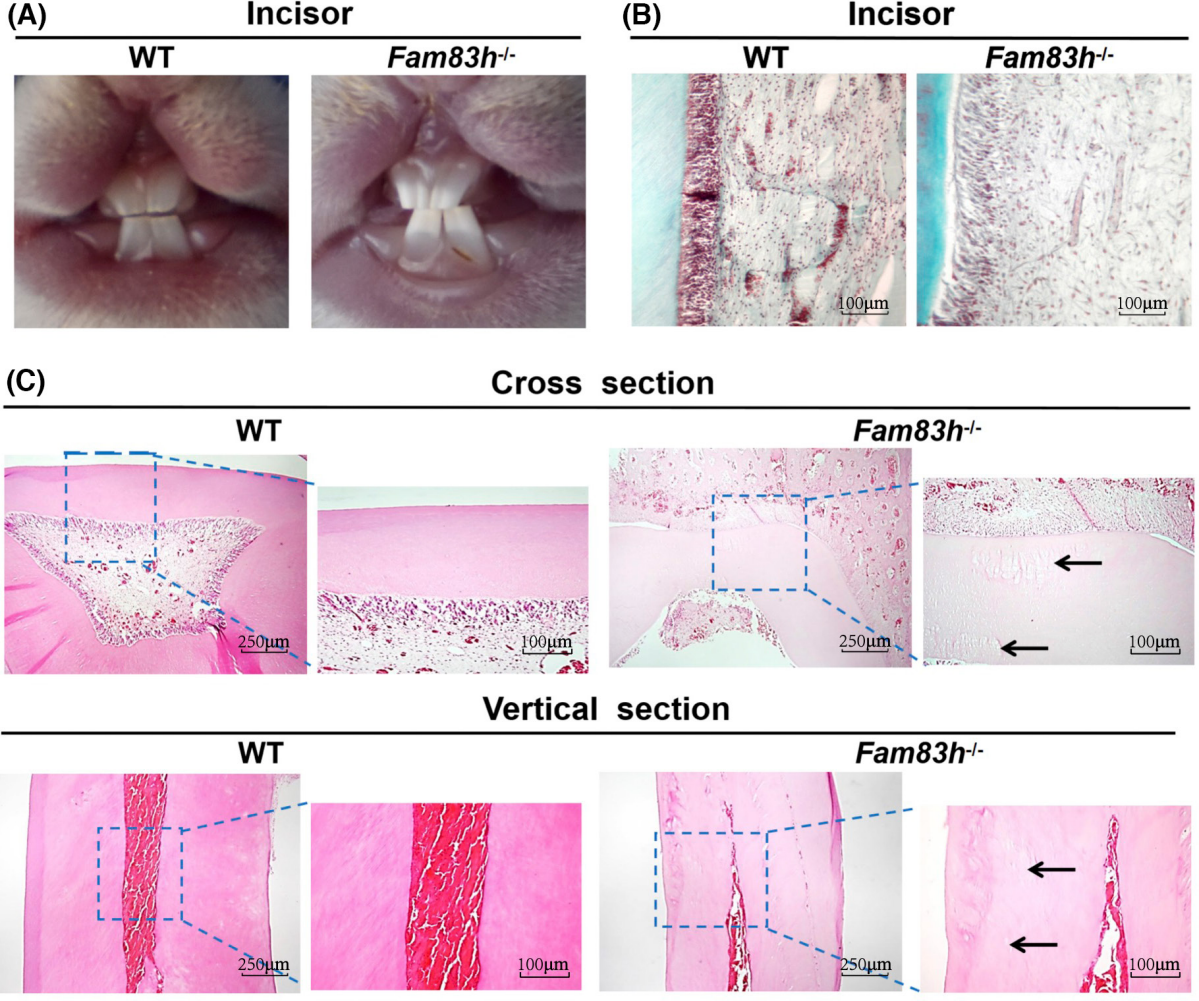
Phenotypic characterization of teeth in Fam83h−/− rabbits. (A) Seven‐day‐old Fam83h−/− rabbits exhibited abnormal dental mineralization. (B) Histological images of the incisor in WT and Fam83h−/− rabbits. Masson‐stained dental pulp cells (red arrows) were sparse and decreased in Fam83h−/− compared with WT rabbits. (C) Histological images of the upper lateral incisors in WT and Fam83h−/− rabbits. The dentine of fifteen‐month‐old Fam83h−/− rabbits showed osteoporosis (black arrows) and reduced erythrocyte numbers in dental pulp cavity. The black arrows identify osteoporosis. The blue frame represents the enlarged area. WT, wild‐type control; *Fam83h*−/−: homozygous *Fam83h* knockout rabbits. Scale bar, 100 μm, 250 μm

To determine the effect of *Fam83h* KO on the formation of dental enamel, we compared the tooth morphology of F2 *Fam83h* ^−/−^ and WT littermate rabbits. Abnormal dental mineralization (Figure 2A) and a decrease in dental pulp cells (Figure 2B) were observed in 7‐day‐old *Fam83h* ^−/−^ rabbits but not in WT littermates. In addition, evidence of osteoporosis and reduced red blood cells numbers was also detected in the dentine of *Fam83h* ^−/−^ rabbits compared with the WT control (Figure 2C). Furthermore, normal morphology and enamel of the molars were determined in *Fam83h* ^−/−^ rabbits, which is consistent with the previously study reported in *Fam83h* null mice^2^, ^14^ (Figure S1).

### 3.3 Abnormal hair cycling in *Fam83h* ^−/−^ rabbits

**FIGURE 3:**
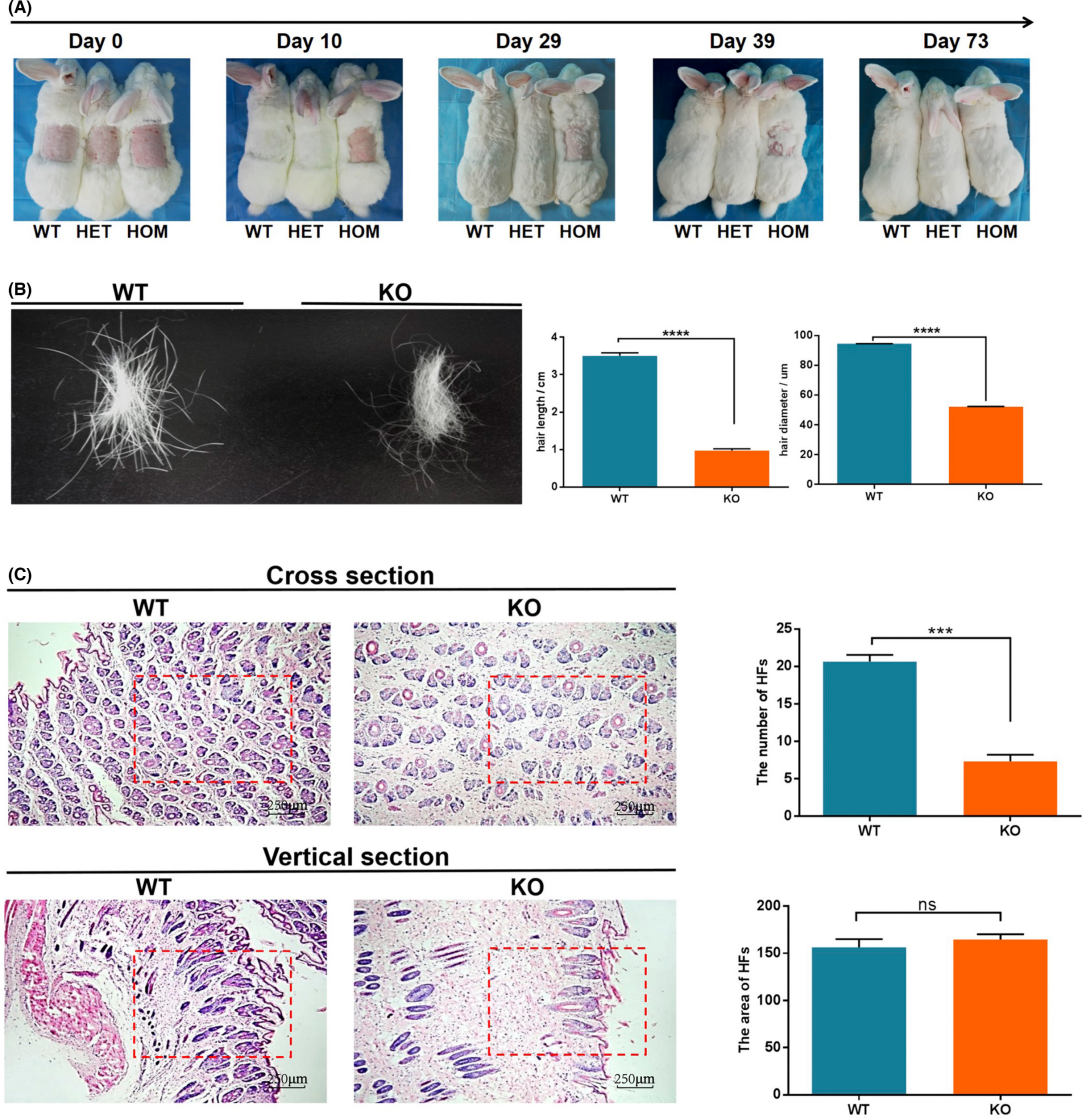
Phenotypic characterization of hair in *Fam83h*−/− rabbits. (A) The speed of hair regrowth in WT and knockout rabbits by photo. Delayed hair cycling in *Fam83h* knockout rabbits. (B) Length measurement of regrowth hair in WT and *Fam83h*−/− rabbits. The regrowth hair in *Fam83h*−/− rabbits were shorter and slower than WT rabbits after shaving 73 days. Regrowth hair of *Fam83h*−/− rabbits exhibited finer diameter than WT rabbits. (C) Histological and statistical analysis of the mean number of hair follicles of the WT and *Fam83h*−/− rabbits. The number of hair follicles were decreased in the mid‐dorsal region of *Fam83h*−/− rabbits by H&E stain. HET: heterozygous *Fam83h* knockout rabbits; HOM: homozygous *Fam83h* knockout rabbits; WT: wild‐type control. All experiments were repeated for three times for each gene. Data are presented as the mean ± SEM and analysed by *t*‐tests using Gnraphpad Prism software 6.0. ***p < 0.001; ****p < 0.0001. Scale bar, 250 μm

A previous study showed that *Fam83h* interacted with the keratin cytoskeleton, and sparse, scruffy coats were determined in *Fam83h* ^**−/−**^ mice.^15^ To identify the skin homeostasis phenotype of the *Fam83h* ^**−/−**^ rabbits, the mid‐dorsal region of WT and *Fam83h* ^**−/−**^ rabbits was shaved at the same day. As shown in Figure 3A, hair regeneration was first identified in WT rabbits after two days of shaving, but it was not detected in *Fam83h* ^**−/−**^ rabbits before 10 days post‐shaving. At 39 days post‐shaving, the obvious hair regeneration was detected in the *Fam83h* ^**−/−**^ rabbits, while 2.146 ± 0.05192 centimetres of hair length was determined in WT rabbits. In fact, the hair of the *Fam83h* ^**−/−**^ rabbits was not completely recovered until 73 days after shaving (Figure 3A). Notably, aside from the decreased rate of hair growth, the hair of *Fam83h* ^−/−^ rabbit was shorter in length and had a finer diameter; the dorsal hair length was approximate 72.17% reduced in *Fam83h* ^**−/−**^ rabbits and the diameter was approximate 44.72% reduced compared with WT controls (Figure 3B). In addition, the density of mid‐dorsal hair follicles in *Fam83h* ^−/−^ rabbit was significantly reduced compared with the WT rabbits (Figure 3C). These results indicate the retarded pelage hair growth and decreased hair follicle density were found in the dorsal skin of *Fam83h* ^−/−^ rabbits.

### 3.4 Defective hair shaft differentiation in *Fam83h* ^−/−^ rabbits

**FIGURE 4:**
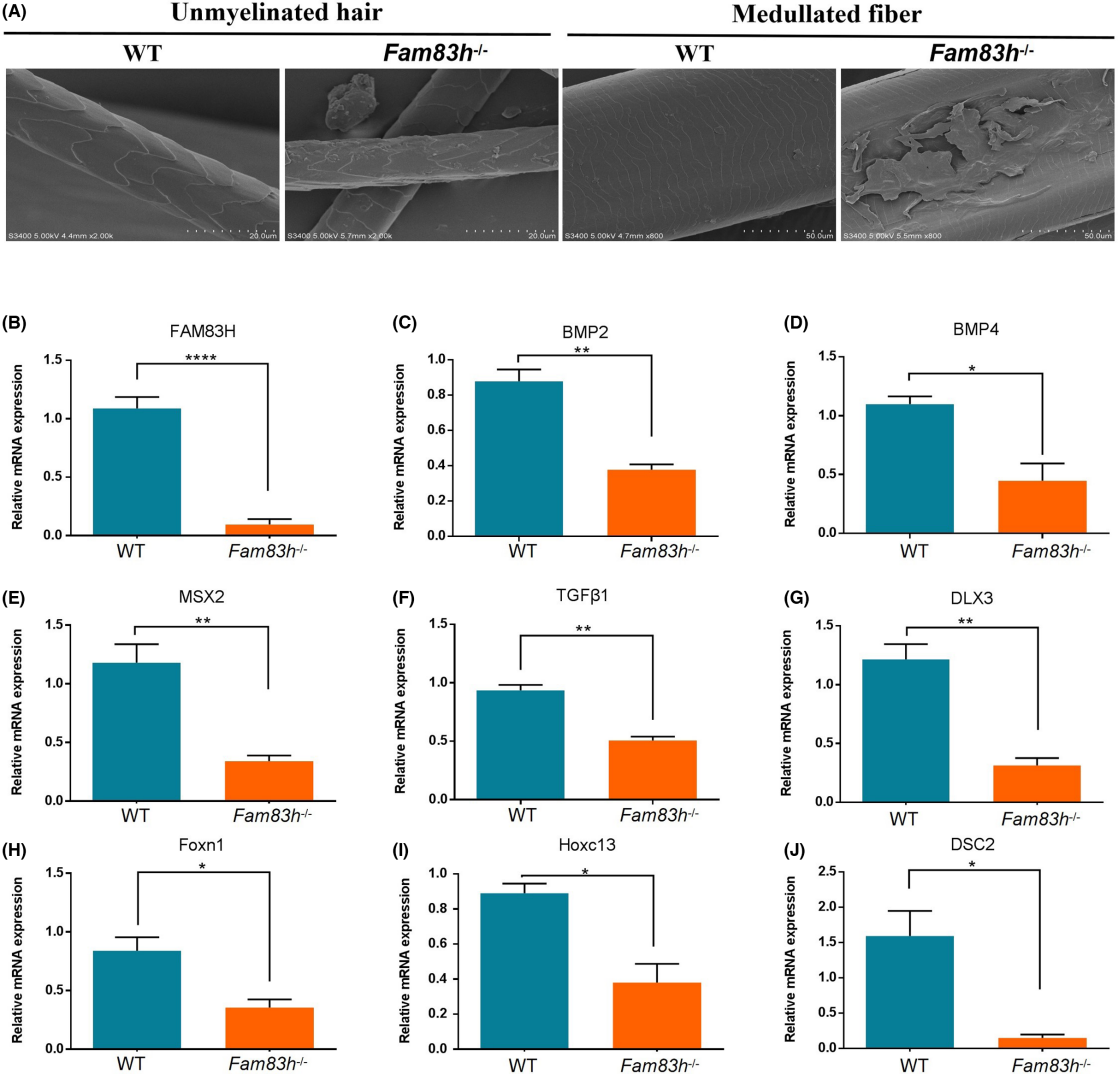
Abnormal hair cycling in *Fam83h*−/− rabbits. (A) Scanning electron microscopy of WT and *Fam83h*−/− rabbits hair fibres shows that unmyelinated hair and medullated fibres were damaged in the mid‐dorsal region of *Fam83h*−/− rabbits. Scale bar, 20 μm and 50 μm. WT: wild‐type control; *Fam83h*−/−: homozygous *Fam83h* knockout rabbits. (B–G) Statistical analysis of the mRNA levels in WT and *Fam83h*−/− rabbits. The mRNA levels of *Fam83h* and bone morphogenetic proteins, acidic hair keratin and hair cuticle, including Bmp2 (C), Bmp4 (D), Msx2 (E), Tgfβ1 (F), Dlx3 (G), Foxn1 (H), Hoxc13 (I) and Dsc2 (J), were markedly reduced in the skin tissues of *Fam83h*−/− rabbits compared with those from in WT rabbits. Dsc, desmosomal cadherin. All experiments were repeated for three times for each gene. Data are presented as the mean ± SEM and analysed by *t*‐tests using Graphpad Prism software 6.0. *p < 0.05; **p < 0.01; ****p < 0.0001

Since *Fam83h* ^**−/−**^ rabbits exhibited shorter and finer hair than WT rabbits, we speculated that the knockout of *Fam83h* impaired hair shaft structure. To identify changes in the hair shaft, hair fibres from the mid‐dorsal region were examined by scanning electron microscope analyses. The result showed the damaged hair cuticle layer in unmyelinated hair and medullated fibres, which exhibited irregular cuticular septation, squamous exuviation and longitudinal fissures on the surface of the hair (Figure 4A) in *Fam83h* ^**−/−**^ rabbits.

Moreover, qPCR was also performed to assess the levels of acidic hair keratin and desmosomal cadherin in *Fam83h* ^**−/−**^ rabbits. The results shown the significantly decreased expression of *Fam83h* (Figure 4B), the hair growth cycle associated genes (BMP2, BMP4, MSX2 and FOXN1),^16^, ^17^, ^18^, ^19^ the hair shaft development associated genes (TGFΒ1, DLX3, FOXN1, HOXC13 and DSC)^19^, ^20^, ^21^, ^22^ in the dorsal skin of *Fam83h* ^**−/−**^ rabbits, which compared with those WT controls (Figure 4C‐J). In conclusion, a novel phenotype of hair cycle abnormalities and hair stem damage caused by *Fam83h* knockout was identified in rabbits for the first time.

### 3.5 Bone abnormalities of *Fam83h* ^−/−^ rabbits

**FIGURE 5:**
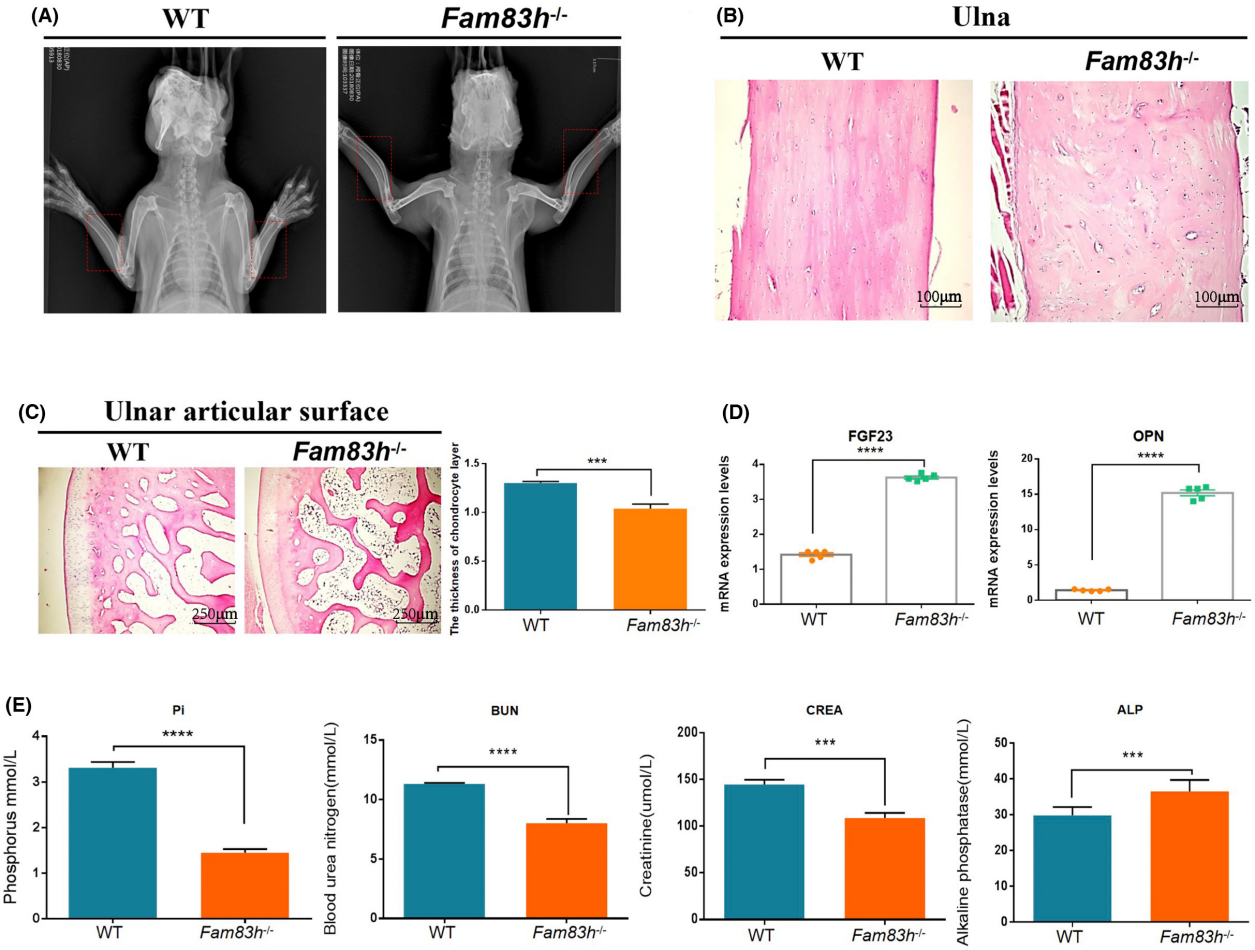
Phenotypic characterization of bone in *Fam83h*−/− rabbits. (A) X‐ray absorptiometry of the ulna and radius in WT and *Fam83h*−/− rabbits. The ulna and radius of fifteen‐month‐old *Fam83h*−/− rabbits exhibited abnormal bending. The red frame represent the bone abnormalities area. (B) Pathological analysis showed that the ulnas of *Fam83h*−/− rabbits were inhomogeneous. (C) The trabecular bone of the ulnar articular surface were reduced in *Fam83h*−/− rabbit by H&E‐stain. (D) Statistical analysis of the mRNA levels in WT and *Fam83h*−/− rabbits. FGF23 and OPN mRNA levels were increased in the femurs of *Fam83h*−/− rabbits. (E) Biochemical analysis of serum from WT and *Fam83h*−/− rabbits. Pathological analysis showed that the kidney medulla of *Fam83h*−/− rabbits had phosphate calcium deposition by von Kossa staining. The level of serum phosphorus, ALP, creatinine, and BUN were significantly elevated in *Fam83h*−/− rabbits. WT: wild‐type control; *Fam83h*−/−: homozygous *Fam83h* knockout rabbits. All experiments were repeated for three times for each gene. Data are presented as the mean ± SEM and analysed by *t*‐tests using Graphpad Prism software 6.0. ***p < 0.001; ****p < 0.0001. Scale bar, 100 μm, 250 μm

To evaluate the effects of *Fam83h* deficiency on bone mineral density and bone morphology, we performed X‐ray autoradiography and histological assessments of the *Fam83h* ^**−/−**^ and WT rabbits. X‐ray autoradiography revealed abnormal bending in the ulna and radius of *Fam83h* ^**−/−**^ rabbits (Figure 5A). Additionally, H&E staining revealed structural inhomogeneity in the ulna of the *Fam83h* ^**−/−**^ rabbits (Figure 5B). Moreover, we found abnormalities in the bone microarchitecture; the decreased number of trabeculae, and the thickness of the cartilage layer was reduced on the ulnar articular surface of the *Fam83h* ^**−/−**^ rabbits (Figure 5C).

Furthermore, qPCR evidence showed that the expression of FGF23 and OPN, regulator of phosphate homeostasis^23^ and inhibitors of mineralization,^24^ were significantly increased in the femurs of *Fam83h* ^−/−^ rabbits (Figure 5D). Moreover, phosphate (Pi), blood urea nitrogen (BUN) and creatinine (CREA) were decreased; showing a negative phosphate balance that impeded skeletal growth in *Fam83h* ^−/−^ rabbits compared with WT rabbits. In contrast, the level of alkaline phosphatase (ALP) was elevated in *Fam83h* ^−/−^ rabbits (Figure 5E), showing disordered phosphate homeostasis. These serological index changes resemble the typical features of human disease, indicating that bone function was affected by phosphate metabolism in *Fam83h* ^−/−^ rabbits. To investigate whether the abnormal femur quality in Fam83h−/− rabbits is influenced by other factors, a histological analysis of the femoral bone marrow was performed. The results showed that no difference in bone marrow of femur between WT and Fam83h−/− rabbits (Figure S3).

## 4 DISCUSSION

The *Fam83h* is associated with intracellular molecular transport,^25^ cytoskeletal network regulation and enamel formation.^6^ To date, the *Fam83h* mutation has been implicated in most autosomal dominant hypocalcified amelogenesis imperfecta (ADHCAI, OMIM *130900) cases,^7^ which are characterized by malformed and easily disrupted enamel following tooth eruption.^1^, ^8^ Although *Fam83h* is known to play an important role in the formation of enamel, the underlying mechanism has not yet been elucidated in detail.

Previous studies showed that most of AI patients exhibited yellow‐brown teeth, localized enamel defects and sustained defective mineralization;^9^ The *Fam83h* mutated mice showed defective dental development and discoloured incisors, even, sparse coat and premature deaths; In this study, the *Fam83h* ^−/−^ rabbits appeared multisystemic lesions, which included abnormal tooth mineralization, defective hair follicle and shaft development. Thus, the comparation of *Fam83h* mutated mice, rabbits and AI humans regarding the phenotypes were listed in Table S4.

Previous studies showed that human dental pulp is a highly vascular tissue that has an highly regenerative capacity due to its unique blood supply.^26^ And, the presence of red blood cells could result in shifts of microbial communities of organisms within the root canal system.^27^ In other words, the normal development of pulp cavity required a number of red blood cells, therefore, we speculated that the reduced red blood cells in incisors will induce the poor nutritional supplied for tooth development of *Fam83h* ^−/−^ rabbits. Consistent with the findings from previously reported, the incisors of *Fam83h* null mice showed a smaller cross‐sectional area of pulp chamber than the other genotype at comparable levels.^2^

Furthermore, the similar lifespan and reproductive ability between *Fam83h* knockout and WT rabbits, which is different in *Fam83h* null mice dying at approximately 2 weeks and generally weak in *Fam83h*‐knockout/lacZ‐knockin mice.^2^ In addition to abnormal dental mineralization, *Fam83h* ^**−/−**^ rabbits exhibited abnormal bone bending and disordered phosphate homeostasis, especially the upregulation of ALP and the downregulation of inorganic phosphate, and the phosphate calcium deposition in kidney medulla of *Fam83h* ^−/−^ rabbits. This is consisted with previous study showing that a *Fam83h* mutation inhibited mineralization via the Wnt/β ‐ catenin signalling pathway.^22^, ^28^

A recent study reported that *Fam83h* KO mice had a slightly scruffy coat,^2^ suggesting that *Fam83h* plays a pivotal role in skin homeostasis.^2^, ^6^ In addition, high expression of *Fam83h* in epithelial cells (included tongue epithelium), suggesting potential functions of *Fam83h* in epithelial cells.^2^, ^5^ Thus, a shorter filiform papilla, a finer myofiber and a thinner stratified squamous epithelium layer were also determined in the *Fam83h* ^−/−^ rabbits in this study (Figure S2). Furthermore, the disorganization of the keratin cytoskeleton by a truncation mutation of *Fam83h* impaired the formation of desmosomes and *Fam83h* affects keratin cytoskeleton formation by recruiting casein kinase I (CK‐1) to keratin,^3^ which play an important role in desmosome formation.^6^ This hypothesis was confirmed in the *Fam83h* ^*−/−*^ rabbits, showing the defects in hair cycling and hair shaft differentiation.

As previously reported, the BMP2/BMP4/MSX2/FOXN1 acidic hair keratin pathway was involved in hair cycling.^29^ In this study, the expression of BMP2, BMP4, MSX2, DLX3, FOXN1, HOXC13 and TGFΒ1 was significantly reduced in *Fam83h* ^*−/−*^ rabbits.^7^, ^13^, ^17^, ^30^ Therefore, we speculated that the *Fam83h* regulates the development of hair, skin and teeth by regulating the expression of the acidic hair keratin genes.^2^, ^13^, ^20^ At present, the role of *Fam83h* in the hair growth cycle and in hair shaft differentiation is still unclear. Thus, further investigation about the function of *Fam83h* in ameloblast differentiation and cytoskeleton network homeostasis are need in the following study.

In summary, this is the first report of a rabbit *Fam83h* ^**−/−**^ model that shows dental defects similar to what is observed in AI: defective hair cycling, hair shaft differentiation and abnormal bending of the ulna and radius. This novel *Fam83h* knockout rabbit model may facilitate understanding the function of *Fam83h* and the pathogenic mechanism of AI and skin homeostasis.

## AUTHOR CONTRIBUTIONS

**Yuning Song:** Formal analysis (equal); funding acquisition (equal); writing – original draft (equal); writing – review and editing (equal). **Yuxin Zhang:** Conceptualization (equal); data curation (equal); methodology (equal); writing – original draft (equal); writing – review and editing (equal). **Jie Yang:** Formal analysis (equal); investigation (equal); validation (equal). **Haobin Yao:** Data curation (equal); investigation (equal); project administration (equal). **Zhongtian Zhang:** Investigation (equal); writing – original draft (equal); writing – review and editing (equal).

## CONFLICT OF INTEREST

No conflict of interest to disclose.

## Supporting information


Figure S1‐S3

Table S1‐S4

## Data Availability

The data that support the findings of this study are available from the corresponding author upon reasonable request.
