# Supplementary material for: CRISPR/Cas9‐mediated deletion of Fam83h induces defective tooth mineralization and hair development in rabbits
Source: J Cell Mol Med. 2022 Oct 27;26(22):5670–9. doi: 10.1111/jcmm.17597 (PMC9667525; doi:10.1111/jcmm.17597)
Supplement: Supplementary file 1 — Figure S1‐S3 [file JCMM-26-5670-s001.pdf]

Supplementary Data

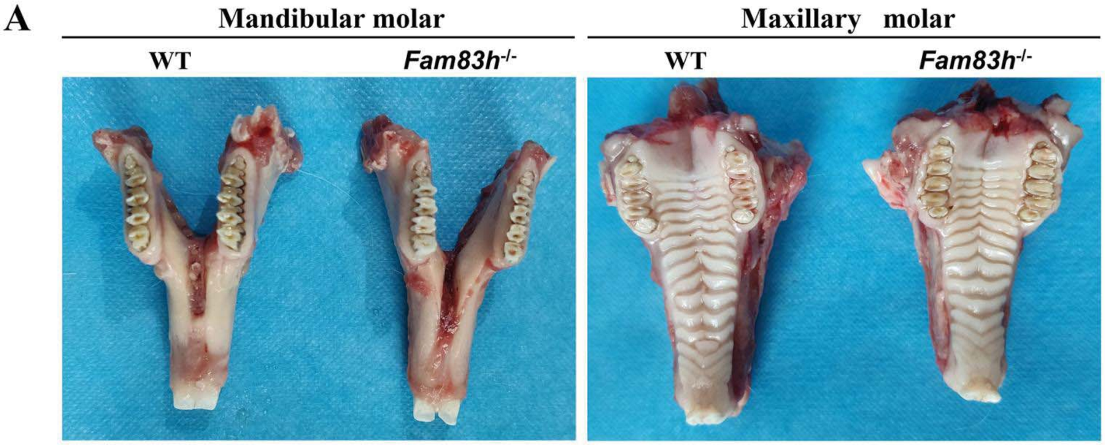

Figure S1. The molars of WT and *Fam83h*$^{-/-}$ rabbits.

(A) The normal size and morphology of molars (fifteen-month-old). WT: wild-type control; *Fam83h*$^{-/-}$: homozygous Fam83h knockout rabbits.

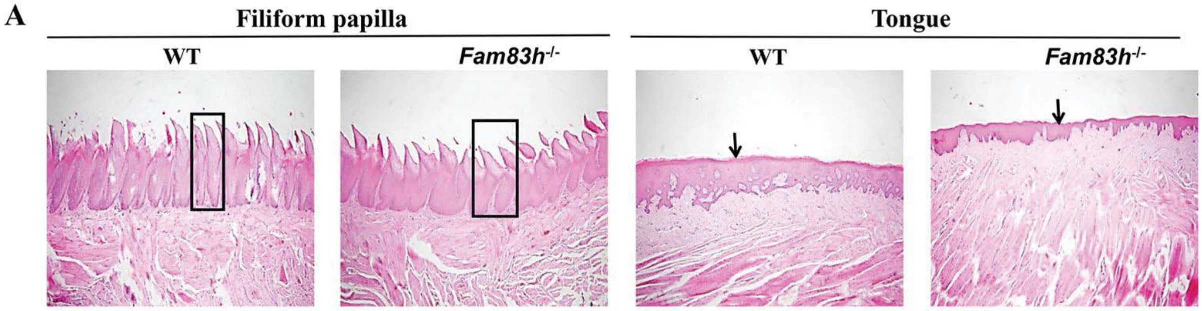

Figure S2. Histological analysis of the glossal epithelial tissue.

A. The shorter tongue filiform papillae and thinner tongue stratified squamous epithelium in *Fam83h*$^{-/-}$ rabbits, which compared with WT controls. WT: wild-type control; *Fam83h*$^{-/-}$: homozygous *Fam83h* knockout rabbits.

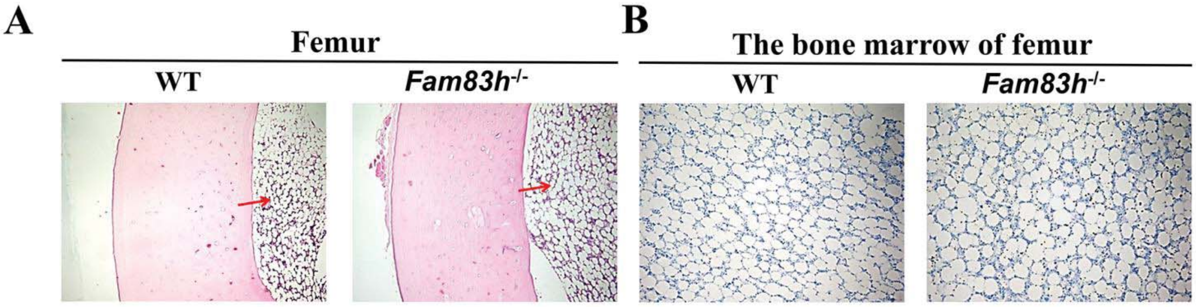

Figure S3. Histological analysis of the bone marrow in femur.

(A) H&E-stained cross sections of femoral bone marrow in *Fam83h*$^{-/-}$ rabbits. (B) There is no difference in bone marrow of femur between WT and *Fam83h*$^{-/-}$ rabbits. The red arrows represent the bone marrow of femur area. The blue ovals represent the femur cavitation in *Fam83h*$^{-/-}$ rabbits. WT: wild-type control; *Fam83h*$^{-/-:}$ homozygous *Fam83h* knockout rabbits.
