# Supplementary material for: CRISPR/Cas9‐mediated deletion of Fam83h induces defective tooth mineralization and hair development in rabbits
Source: J Cell Mol Med. 2022 Oct 27;26(22):5670–9. doi: 10.1111/jcmm.17597 (PMC9667525; doi:10.1111/jcmm.17597)
Supplement: Supplementary file 2 — Table S1‐S4 [file JCMM-26-5670-s002.pdf]

Supplementary Table

Table S1. Primers used for construction of sgRNA expression plasmids and PCR mutation detection.

NO.	Name	Primers	Sequence(5'-3')
1	sgRNA1	gRNA-1-F	TAGGCTGGGCGAACGAGTCGCGC
		gRNA-1-R	AAACGCGCGACTCGTTCGCCCAG
2	sgRNA2	gRNA-2-F	TAGGCTTCGAGGTGTTCTGCAAG
		gRNA-2-R	AAACCTTGCAGAACACCTCGAAG
3	*Fam83h*	*Fam83h*-F	CACAGCAAGGCTGTCGTGTCC
		Fam83h-R	GAACTTGCCCACCTTGCTGTC

Table S2.The primers of potial off-target sites(POTS) used in this study

	Potential Off Target Site	Position	PCR Primer
**S1**	CTGGGCGAGCGAGCAGCGCCGG	chr10:+29463027	F:TGTCTTTGCTTTGTGGACCTA<br>R:GATGGATCACCACTCTCATTCA
	CTGGGCCACCAAGTCGCGCCGG	Chr13:-116258003	F:CCCGGAATCTCCTTTCCTTTC<br>R:CCTCCCAGGAGGCTAAGAA
	TTCGACACTCGCTCGCCCAGCAG	chr8:+51862195	F:GTACAGTACGTGTCTAGCGAAG<br>R:TGAGAGAACCGAATGAGTCAAA
	CCGGGCGAGCGGGTCGCGCGGG	Chr15:-23393164	F:CTTGCACGCACACATTCTAC<br>R:GGTGTACCATTCCTCCTTCTC
	TGCGCGGGGCCTTCGCCCAGGGG	chr7:+114352893	F:GGGACTCAGAGTTGGATTTCTT<br>R:GTCTTCAGATCGCCGTCATT
**S2**	GCTGGGATGTGTTCTGCAAAGGG	chrUN0:-134434	F:AGTGACTTGCACGGACTTAC<br>R:GTGTTCTCAGGAAGAAGGAGTG
	GCAGTGAAGTGTTCTGCAAGTGG	chr9:-9723322	F:AAGGGTTCCTCGTCCTATGT<br>R:CCTGGATTGTTCACTGGATACC
	GGTGCGGAGTGTTCTGCAAGAGG	chrUN0:+18468	F:AGCCTGGGACTGAGAAAGT<br>R:TGCACAAGGCTCTGGAATG
	GCTTAGCCGGGTTCTGCAAGGGG	chr19:+44051549	F:CTTAGGTCTTCCTGCCAACTC<br>R:TGCAGTGATTGTCCTCATCC
	GCTTTGATGACTTCTGCAAGTGG	chrUN0:+97155	F:CAGAGAGTGATCGAGCAAAGAG<br>R:GTGGAAGGGTCAGAACTCAAA

TableS3. Primers used for quantitative real - time RT-PCR

Name	Primers	Sequence（5’-3’）
GAPDH	GAPDH-F	TTCCACGGCAGGTCAAGGC
	GAPDH-R	GGGCACCAGCATCACCCCAC
FGF23	FGF23-F	CCTGCAGATCCACAAGGAC
	FGF23-R	ACACCTGTTATCACCACGAAG
OPN	OPN-F	GCTAAACCCTGACCCATCTC
	OPN-R	GTCATGGCTTTCAATGGACTTAC
BMP2	BMP2-F	CACCCGGCGATTCTTCTTTA
	BMP2-R	AGCTTCCTGCATCTGTTCTC
BMP4	BMP4-F	CGGATTACATGCGGGATCTTTA
	BMP4-R	CAGATGTTCTTCGTGGTGGAA
MSX2	MSX2-F	TCGGTCAAGTCGGAGAACT
	MSX2-R	CGATTGGTCTTGTGCTTCCT
DLX3	DLX3-F	CGTTTCCAGAAGGCACAGTA
	DLX3-R	CCCGTTCTTGTAGAGCTTCTT
FOXN1	FOXN1-F	GCCACAGTCTGACGTCTC
	FOXN1-R	GTCGGGCATGAATGAGGA
HOXC13	HOXC13-F	ACCTCTGGAAGTCTCCCTT
	HOXC13-R	CTGGCTGCGTACTCTTTCTC
DSC2	DSC2-F	GACCCTACATGTTCCCTCTAAAC
	DSC2-R	GGATCACTTGAATGAATTAGACTTGG
TGFβ1	TGFβ1-F	CTGCAGAGGCTCAAGTTACA
	TGFβ1-R	CTTTGTTGTCACAGGAACAGTG

Table S4. The comparation of *Fam83h* mutated mice, rabbits, and AI humans regarding the phenotypes.

Species	Phenotypes			
	Tooth	Hair	Bone	Others
**Human**	1. Normal thickness, cheesy soft and lost soon after eruption(33)<br>2. Yellow-brown teeth, localized enamel defects, sustained defective mineralization(9).<br>3. Yellow-brown discoloration(34).<br>4. Yellowish brown colouration and rough surface, soft enamel , enamel largely missing from the crown(35).<br>5. Yellow to brownish discoloration , partial or complete loss of enamel , irregular rough tooth surfaces, heavy plaque accumulation and gingivitis(36).<br>6. Extensive discoloration and degradation of enamel, extensive posteruptive enamel loss and discoloration of the remaining enamel(37).<br>7. Yellowish to brownish discoloration, irregular rough surface, enamel loss, hypocalcified enamel(38).<br>8. Soft, uncalcified enamel(39).	**No report**	**No report**	**No report**
**Mice**	1.Glossy and smooth enamel surfaces and sharp incisal tips, with normal crown morphology, enamel thickness, surface texture, smaller cross-sectional area of pulp chamber, mild alveolar bone destruction(2).<br>2. Decrease in the enamel surface hardness(40).<br>3. Defect in dental development and the enamel formation , growth retardation and discolored incisors that were prone to breakage and erosion after breastfeeding in mandible incisor(29).<br>4. Enamel malformations, thinner incisor enamel layer , surface roughness and altered enamel rod orientation(14)	1. Expression in the epithelial tissues （notably in skin and hair follicles）which manifested a disease phenotype(2).<br>2. Sparse, scruffy coat(29).	**No report**	1.Died after 2 weeks and rarely survived to 7 weeks, smaller body size, reduced general activity(2).<br>2.Small body size and decreased general activity(29), delay in opening the eyes and dry eyes(29).
**Rabbits**	1. Abnormal tooth mineralization and loose dentine*.	1. Reduced hair follicle counts of dorsal skin#<br>2. Hair cycling dysfunction and hair shaft differentiation deficiency#.	1. Abnormal bending in ulna and radius#	**No report**

*, predicted phenotypes; #, novel phenotypes.
